# The presence of the recurrent veinlet in the Middle Jurassic Nymphidae (Neuroptera): a unique character condition in Myrmeleontoidea

**DOI:** 10.3897/zookeys.325.5453

**Published:** 2013-08-20

**Authors:** Vladimir N. Makarkin, Qiang Yang, Chaofan Shi, Dong Ren

**Affiliations:** 1 College of Life Sciences, Capital Normal University, 105 Xisanhuanbeilu, Haidian District, Beijing, 100048, China; 2 Institute of Biology and Soil Sciences, Far Eastern Branch of the Russian Academy of Sciences, 100 let Vladivostoku Avenue 159, Vladivostok, 690022, Russia

**Keywords:** Neuroptera, Nymphidae, recurrent veinlet, subcosta anterior, Daohugou, Middle Jurassic

## Abstract

A well-developed recurrent veinlet is found in the forewing of two species of Nymphidae from the Middle Jurassic locality of Daohugou (Inner Mongolia, China), *Liminympha makarkini* Ren & Engel and *Daonymphes bisulca* **gen. et sp. n.** This is the first record of this trait in the clade comprised of the superfamilies Myrmeleontoidea and Chrysopoidea. We interpret the recurrent veinlet in these species as a remnant of the condition present more basally in the psychopsoid + ithonoid + chrysopoid + myrmeleontoid clade (i.e., as a plesiomorphy). Other venational character states of *Daonymphes bisulca* of interest include the configuration of subcosta anterior (ScA), which is very similar to that of extant Nymphidae. We consider the short ScA terminating on ScP to be an autapomorphy of Neuroptera.

## Introduction

Understanding the evolution of particular morphological characters during the broad historical development of higher insect taxa is a very interesting and important question. In fact, the phylogeny of a taxon may be realistic only if the evolutionary transformations of all available characters are correctly interpreted (Hennig 1981; Bechly 2000; Kukalová-Peck 2008).

The forewing humeral veinlet (i.e., the proximal-most veinlet of ScP) in all Neuropterida (and generally in all Neoptera) is plesiomorphically a simple, crossvein-like. However, in some families of Neuroptera it is curved, obviously directed towards wing base (i.e., recurrent), and often profusely branched. This condition (the recurrent veinlet for short) is one of the significant informative venational characters found within this order. It is characteristic of several families and subfamilies, the vast majority of which have the costal space basally dilated (see below). However, it was hitherto not found in any of numerous species of the clade comprised the superfamilies Myrmeleontoidea (Nymphidae, Nemopteridae, Ascalaphidae, Myrmeleontidae, Palaeoleontidae and Babinskaiidae) and Chrysopoidea (Chrysopidae, Mesochrysopidae and Ascalochrysidae) (Yang et al. 2012: fig. 32). The costal space in the vast majority of species of this clade is basally narrowed.

Nymphidae are generally accepted to be the most basal family in the superfamilies Myrmeleontoidea, and the only myrmeleontoid family known from the Jurassic. In general, 18 fossil (named) species are known in the family (Krüger 1923; Carpenter 1929; Panfilov 1980; Makarkin 1990a, b; Ponomarenko 1992a; Martins-Neto 2005; Menon et al. 2005; Ren and Engel 2007; Engel and Grimaldi 2008; Archibald et al. 2009; Jepson et al. 2012; Shi et al. in press). Here, we report the occurrence of a recurrent veinlet in the two oldest known specimens of Nymphidae, recovered from the Middle Jurassic Daohugou locality, China: *Liminympha makarkini* Ren & Engel, 2007; and a new species, described here. Three other nymphid specimens are known from that locality; the recurrent veinlet appears to be absent in one (but its humeral area is poorly preserved), and the basal portion of wings is missing in other two (Shi et al. in press). In this paper, we re-describe *Liminympha makarkini*, describe a new genus and species, and discuss the occurrence of the recurrent veinlet in neuropteran taxa and its possible phylogenetic implications. Also, we discuss the presence of the distinct subcosta anterior in *Daonymphes bisulca* gen. et sp. n. We consider ScA terminating on ScP to be an autapomorphy of Neuroptera.

## Material and methods

This paper is based on two specimens collected from the Daohugou locality and housed in the Key Laboratory of Insect Evolution and Environmental Changes, College of Life Sciences, Capital Normal University, Beijing, China (CNUB; Dong Ren, curator). Daohugou Village is situated in Shantou Township, Ningcheng County, Inner Mongolia, China. The insect-bearing beds of the Daohugou locality are considered to belong to the Jiulongshan Formation, and are dated as Bathonian, Middle Jurassic (Gao and Ren 2006) or Bathonian to Callovian (Middle to late Middle Jurassic) (Yang et al. 2012).

The specimens were examined under a Leica MZ12.5 dissecting microscope; line drawings were prepared with CorelDraw 12 graphics software and Adobe Illustrator CS5 with the aid of Adobe Photoshop CS3; photographed by a Nikon SMZ1000 stereomicroscope; the whole specimens were photographed by Nikon D100 Digital Camera.

**Terminology and abbreviations.** Wing venation terminology in general follows Kukalová-Peck and Lawrence (2004), except that we treat the median vein as in Yang et al. (2012). Terminology of wing spaces and details of the venation (e.g., subcostal veinlets) follows Oswald (1993).

**Venational abbreviations.** AA analis anterior; AP analis posterior; Cu cubitus; CuA cubitus anterior; CuP cubitus posterior; M media; MA media anterior; MP media posterior; R radius; RA radius anterior; RP radius posterior; RP1 proximal-most branch of RP; rv recurrent veinlet; ScP subcosta posterior.

## Systematic paleontology

### Order Neuroptera Linnaeus, 1758
Family Nymphidae Rambur, 1842

#### Genus Daonymphes gen. n.

http://zoobank.org/17B6B9EB-D7C9-46A1-A8F9-0F5631FDA7B7

http://species-id.net/wiki/Daonymphes

##### Type and only species.

*Daonymphes bisulca* sp. n.

##### Diagnosis.

Forewing broad proximally [strongly narrowed proximally in *Liminympha*]; vast majority of proximal subcostal veinlets forked [simple in all other Mesozoic genera]; crossveins between branches of MP absent [present in *Nymphites* Haase, 1890, *Mesonymphes* Carpenter, 1929]; CuP space broad, nearly two times as wide as intracubital space [nearly as wide as intracubital space in *Liminympha*].

##### Etymology.

From Daohugou, the locality of the type species, and *Nymphes*, a genus-group name. Gender: feminine.

##### Remarks.

This genus is most closely related to four other Mesozoic genera (*Nymphites*, *Mesonymphes*, *Liminympha* and *Sialium* Westwood, 1854) but clearly distinguished from these as indicated in the diagnosis. The latter genus is only known from single hind wing from the early Berriasian of Purbeck, England. In general, Nymphidae occur very rare in the Jurassic, only six specimens are known. The monotypic genera *Liminympha* (Middle Jurassic [Bathonian/Callovian] of Daohugou, China) and *Mesonymphes* (Late Jurassic [Tithonian] of Solnhofen, Germany) are represented by almost complete specimens possessing both fore- and hind wings. The type species of *Nymphites* from Solnhofen is represented by only two incomplete hind wings. Two species from Daohugou are assigned to this genus: one species (two specimens) have almost complete fore- and hind wings; the other only hind wings (Shi et al. in press).

#### Daonymphes bisulca sp. n.

http://zoobank.org/61D8C637-96AD-4D41-BEF6-270FE5F3A6BA

http://species-id.net/wiki/Daonymphes_bisulca

Figs 1 , 2 , 6A

##### Diagnosis.

As for the genus.

##### Description.

Forewing 29 mm long as preserved (estimated complete length about 40 mm), 11.5 mm wide. Costal space relatively broad for entire length, narrowed basally. Humeral veinlet recurrent, with one short branch. Subcostal veinlets somewhat curved, mostly forked once or twice, shallowly, few deeply; several proximal veinlets simple. ScA well developed, terminating on ScP within humeral area. Subcostal space narrow, with several scarce crossveins. RA space relatively narrow, slightly narrowed towards wing apex, with relatively numerous crossveins. RP with nine preserved branches, becoming more closely spaced towards wing apex. Crossveins rather numerous over entire radial space, irregularly spaced. M forked distal of origin of RP. MA incomplete, simple for entire preserved length. MP distally pectinate, with six preserved branches, most of these forked once or twice; no crossveins detected between branches. Cu divided into CuA, CuP rather far from wing base. CuA distally pectinate, with six preserved branches, all forked once or twice; one series of crossveins between branches of CuA, continued CuP (‘pseudo-CuP’). CuP long, strongly pectinately branched with nine long branches; of four proximal-most branches, two forked; all distal branches dichotomously forked; no crossveins detected. Eleven crossveins between CuA, CuP rather irregularly spaced. AA3+4 and its posterior branch deeply forked. Basal crossvein between CuP, AA3+4 very short; distal crossvein strongly oblique. AP1+2 deeply forked. AP3+4 not preserved. Membrane around one crossvein between MA, MP near MP and three crossveins between CuA, CuP near CuA broadly heavily shaded; most crossveins between RA, RP apparently broadly shaded, pale fuscous.

**Figure 1.**
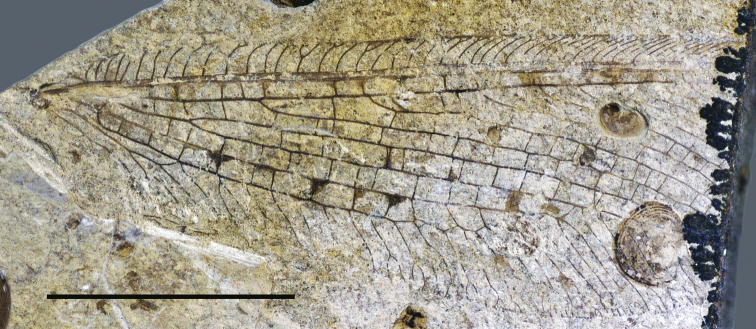
*Daonymphes bisulca* gen. et sp. n. Holotype CNU-NEU-NN2011119 as preserved. Scale bar = 10 mm.

**Figure 2.**
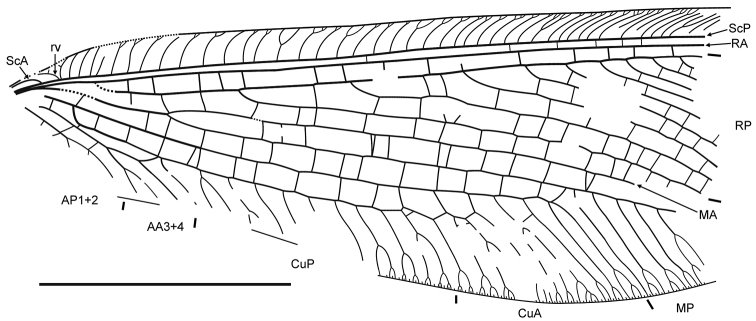
*Daonymphes bisulca* gen. et sp. n. Forewing venation of the holotype CNU-NEU-NN2011119. Scale bar = 10 mm.

##### Material.

Holotype CNU-NEU-NN2011119, deposited in CNUB; an incomplete forewing.

##### Type locality and horizon.

Daohugou Village, Shantou Township, Ningcheng County, Inner Mongolia, China. Jiulongshan Formation, Middle Jurassic.

##### Etymology.

From the Latin *bisulcus,-a,-um*, forked, divided into two parts, in reference to its forked subcostal veinlets.

#### Liminympha

Ren & Engel, 2007

http://species-id.net/wiki/Liminympha

Liminympha Ren & Engel, 2007:212.

##### Type and only species.

*Liminympha makarkini* Ren & Engel, 2007, by original designation.

##### Diagnosis (revised).

Forewing strongly narrowed proximally [broad proximally in *Nymphites*, *Daonymphes* gen. n. and *Mesonymphes*]; all veinlets of ScP simple [at least distal veinlets forked once or twice in *Nymphites*, *Daonymphes* gen. n.]; CuP space narrow, nearly as wide as intracubital space [nearly two times as wide as intracubital space in *Nymphites*, *Daonymphes* gen. n. and *Mesonymphes*]. Hind wing MP with four pectinate branches [eight in *Sialium*]; branches of CuA not connected by crossveins [at least proximal branches of CuA connected by crossveins in *Nymphites*]; anterior branch of CuP simple [deeply forked in *Nymphites* and *Mesonymphes*].

#### Liminympha makarkini

Ren & Engel, 2007

http://species-id.net/wiki/Liminympha_makarkini

Figs 3 – 5 , 6B

Liminympha makarkini Ren & Engel, 2007:212, figs 1–3; Engel and Grimaldi 2008:9; Yang et al. 2010:177.

##### Redescription.

Body (metathorax, abdomen) poorly, fragmentarily preserved. First abdominal tergite rather long; distally with distinct transverse suture, probably heavily sclerotized; medially with mediolongitudinal short suture; portion of 1st tergite distal to transverse suture (‘Transversalnaht’ of Achtelig 1975) very narrow (Fig. 4). Second tergite nearly as wide as long. Other tergites indistinct.

**Figure 3.**
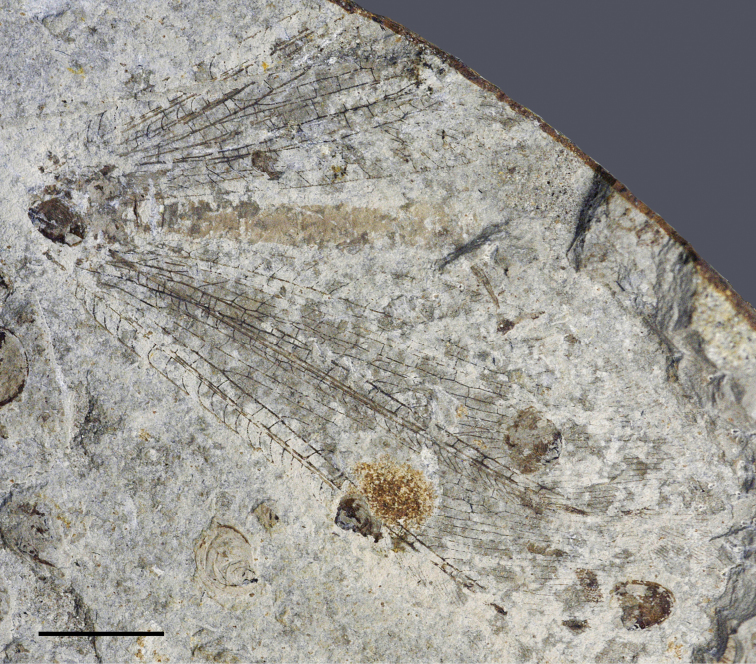
*Liminympha makarkini* Ren & Engel, 2007. Holotype CNU-NEU-NN1999024 (counterpart) as preserved.Scale bar = 5 mm.

**Figure 4.**
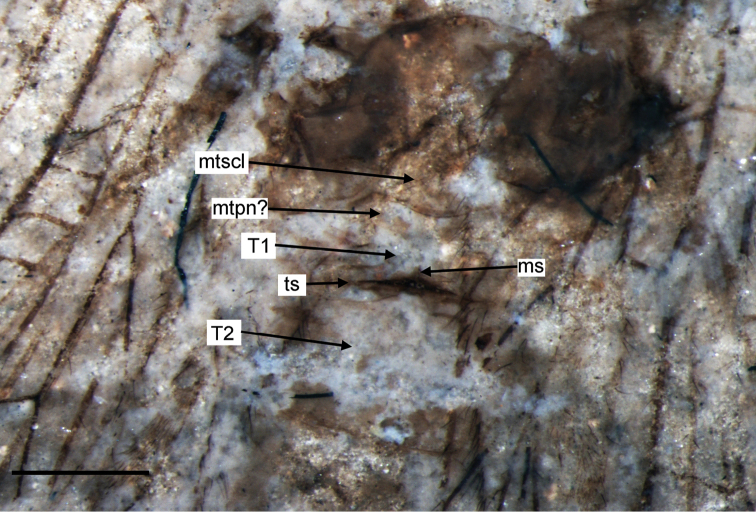
*Liminympha makarkini* Ren & Engel, 2007. Metathorax and the basal part of abdomen of the holotype CNU-NEU-NN1999024 (counterpart). Wetted with ethanol. Abbreviations: **ms**, mediolongitudinal suture of 1st abdominal tergite; **mtpn**, metapostnotum; **mtscl**, metascutellum; **ts**, transverse suture of 1st abdominal tergite; **T1, T2**,1st, 2nd abdominal tergites.Scale bar = 1 mm.

Forewing elongate, narrowed in proximal portion, most dilated at distal 3/4 length; about 30 mm long, 8 mm wide. Costal space narrow, basally narrowed, distally dilated. All preserved subcostal veinlets simple, strongly oblique distally; veinlets of ScP+RA dichotomously branched. Humeral veinlet rather strongly recurrent, branching not detected (Fig. 6B). Subcostal space very narrow; two crossveins in distal part detected, others possible. RA spaces slightly narrower than costal space, narrowed towards apex; crossveins irregularly spaced for entire preserved portion. RP originates rather near wing base, with about 16 branches; RP1 originates far from origin of RP; at least four proximal-most branches widely spaced, more distal branches closely spaced. In right forewing, RP2 terminated at RP1; RP3, RP4 fused for short distance (probably aberrations). Crossveins between branches of RP very scarce, restricted to area between RP1 to RP5. M appears fused with R basally; forked at nearly equal distance from origin of RP, origin of RP1. MA long, slightly arched, distally few-branched. MP long, its anterior trace (stem of MP) nearly straight before terminal branching; with five long distal branches, quite strongly inclined. Crossveins between R/RP and MA, MA and MP irregularly spaced, arranged differently in right, left wings. Cu dividing into CuA, CuP rather near to wing base. CuA long, smoothly curved anteriorly, pectinate with four long branches, each dichotomously branched. Between branches of CuA at last three crossveins forming gradate series continued CuP (‘pseudo-CuP’). CuP long, pectinately branched, with seven-eight rather short branches, most simple. AA3+4 rather short; stem simple, with one or two simple branches. AP1+2 incompletely preserved, probably simple. AP3+4 not preserved. One dark rather big spot in distal portion of radial space might be present (apical half of other wing not preserved).

**Figure 5.**
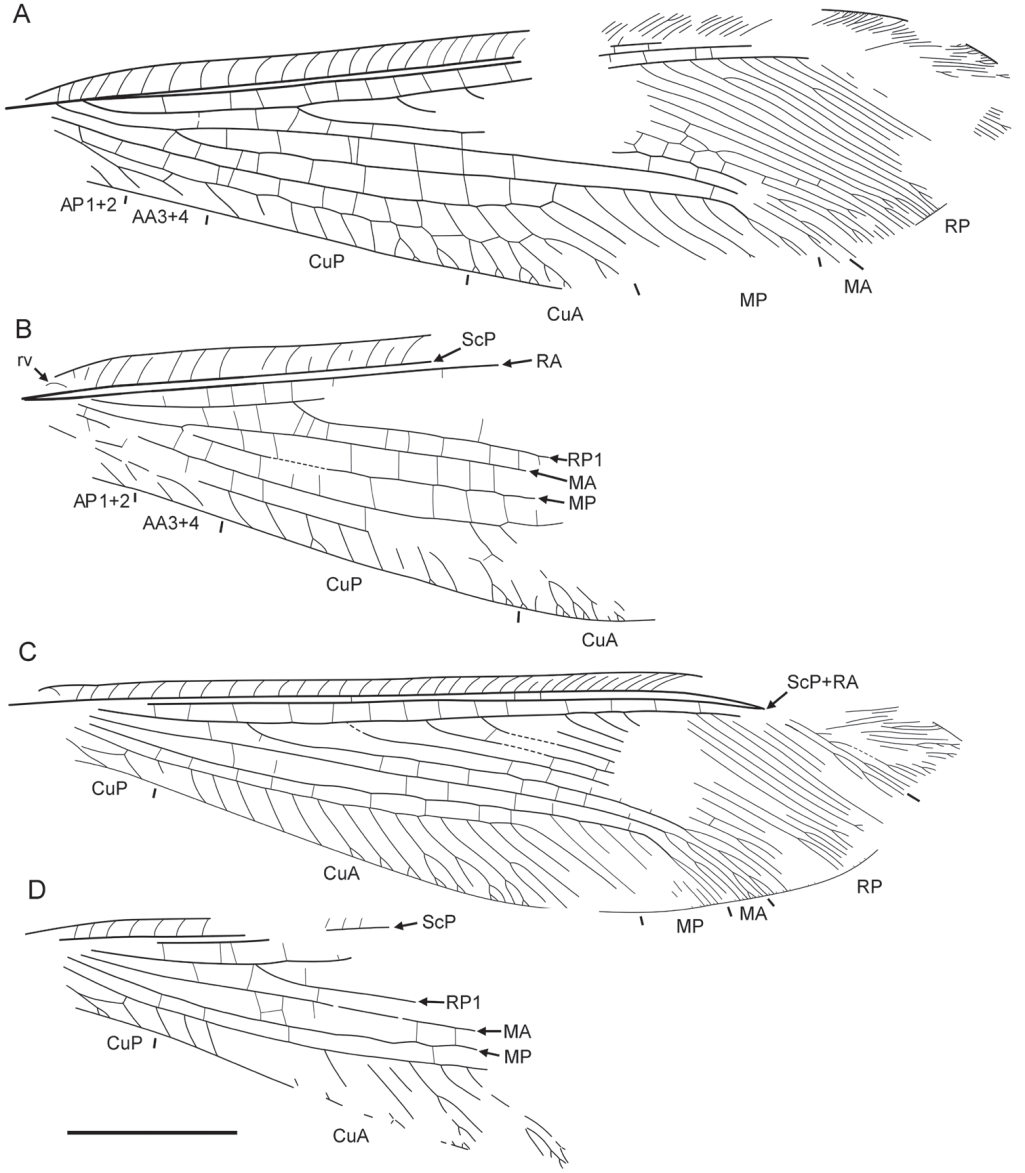
*Liminympha makarkini* Ren & Engel, 2007. Wing venation of the holotype CNU-NEU-NN1999024 **A** right forewing **B** left forewing **C** right hind wing **D** left hind wing. Scale bar = 5 mm (all to scale).

**Figure 6.**
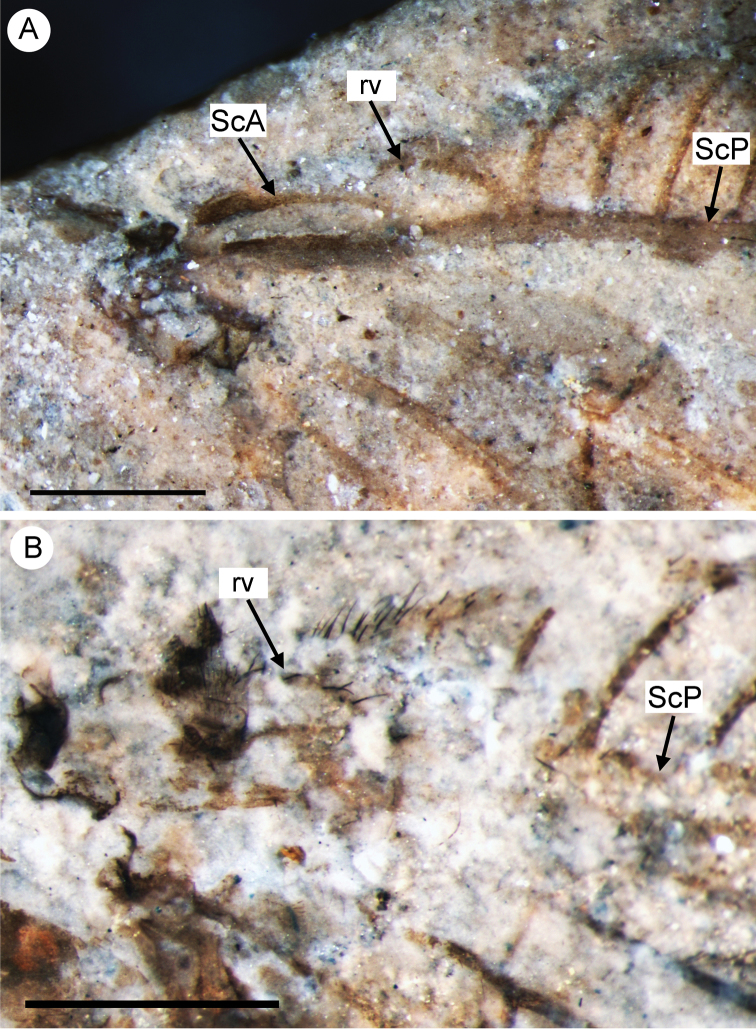
Basal portion of forewings of the Middle Jurassic Nymphidae showing the recurrent veinlet (rv) and the anterior subcosta (ScA) **A** the holotype of *Daonymphes bisulca* gen. et sp. n., CNU-NEU-NN2011119 **B** the holotype of *Liminympha makarkini* Ren & Engel, 2007, CNU-NEU-NN1999024. Both wetted with ethanol. Scale bar = 1 mm.

Hind wing similar in shape to forewing, slightly narrower; about 28 mm long, 7 mm wide. Costal space narrow, dilated distally. Subcostal veinlets simple, becoming more oblique, closely spaced, curved towards apex. Humeral veinlet bent to base, nearly straight, not branched. Subcostal space narrow; three crossveins detected, others possible. ScP, RA fused far from wing apex; preserved veinlets of ScP+RA long, dichotomously forked; crossveins between them not detected. RA space basally as wide as costal space, narrowed towards apex; crossveins rather regularly spaced for entire preserved portion (right wing). RP originated near wing base, with about 15 branches; RP1 originated far from origin of RP (but closer to wing base than in forewing); five proximal-most branches widely spaced, other (distal) branches closely spaced. Crossveins between branches of RP very scarce, restricted to area between RP1 to RP4. Origin of M and its fork not preserved. MA long, nearly straight, distally dichotomously branched. MP long, its anterior trace (stem of MP) slightly incurved, with four long distal branches, quite strongly inclined. Origin of Cu and its dividing into CuA, CuP not present. CuA long, pectinate, with 15 branches, which proximally simple, distally once or twice forked. CuP short, deeply forked. Distal crossvein between CuA, CuP rather short connecting CuA, anterior branch of CuP fork. Anal veins not preserved. Crossveins between R/RP and MA, MA and MP, MP and CuA poorly preserved, irregularly spaced, arranged differently in right and left wings; crossveins between branches of MP, CuA absent.

##### Material.

Holotype CNU-NEU-NN1999024 (part, counterpart), deposited in CNUB; an incomplete specimen.

##### Type locality and horizon.

Daohugou Village, Shantou Township, Ningcheng County, Inner Mongolia, China. Jiulongshan Formation, Middle Jurassic.

##### Remarks.

This redescription is only based on the counterpart of the holotype.

## Discussion

### Recurrent veinlet

The recurrent veinlet is known in the following extant taxa of Neuroptera: all species of Psychopsidae and Ithonidae (including Polystoechotidae and Rapismatidae), the vast majority of Hemerobiidae; rarely in Berothidae (including Rhachiberothinae) and Mantispidae (only Symphrasinae).

The recurrent veinlet has not been detected in any Permian Neuroptera (Permithonidae, Archeosmylidae) and the majority of Triassic taxa represented by these two families, and ‘Mesoberothidae’; however the basal portion of the forewing in any reported specimen of the latter is poorly preserved (e.g., Riek 1955; Lambkin 1988; Vilesov and Novokshonov 1994; Vilesov 1995; Novokshonov 1996; Jell 2004; VM, pers. obs.). In Triassic Neuroptera, the recurrent veinlet is only found in those taxa that have the costal space strongly dilated basally, i.e., the psychopsoids (Psychopsidae, Osmylopsychopidae, and broad-winged taxa whose family affinities are not yet clear). These are: (1) several undescribed Osmylopsychopidae from the Ladinian/Carnian Madygen Formation (Kyrgyzstan) (see Shcherbakov 2008: fig. 6); (2) *Triassopsychops superbus* Tillyard, 1922: fig. 89; (3) *Archepsychops triassicus* Tillyard, 1919: fig. 27; (4) possibly in *Osmylopsychops spillerae* Tillyard, 1923: fig. 93 (however see Lambkin 1992: fig. 2), and (5) *Petropsychops superbus* Riek, 1956: pl. 1, fig. 2 (see also Grimaldi and Engel 2005: fig. 9.15) (all from the Carnian Ipswich Series of Australia). Of these taxa, the recurrent veinlet is most clearly visible in one of the Osmylopsychopidae wings from the Madygen Formation, where it is strongly curved towards the wing base and has eight branches (VM, pers. obs.). In the Australian taxa, the recurrent veinlet is poorly preserved.

Of the Early Jurassic Neuroptera, the recurrent veinlet is detected in all psychopsoids whose forewing base is well preserved (e.g., Tillyard 1933: fig. 2; Ansorge 1996; fig. 52) and in Prohemerobiidae. The latter family, however, remains undefined; we treat it as containing only the type genus *Prohemerobius* Handlirsch, 1906 (probably paraphyletic), recorded from the Early Jurassic of Germany and England (Handlirsch 1906–1908, 1939; Bode 1953; Whalley 1988; Ponomarenko 1995) and the Late Jurassic of Mongolia (Khramov 2011). The majority of species in this genus have the basal portion of the forewing costal space missing or poorly preserved. Still, some species (including the type species *Prohemerobius dilaroides* Handlirsch, 1906) possess the recurrent veinlet, configured similarly to that of the Middle Jurassic Nymphidae, i.e., with few (or one) simple branches (J. Ansorge, VM, pers. obs.). The wings of *Prohemerobius* are not of the psychopsoid type; they are small, relatively narrow with hemerobiid-like venation, and the costal space is narrow compared with those of psychopsoids. All known Early to Middle Jurassic Parakseneuridae possess a very well developed recurrent veinlet, along with up to 15 dichotomously forked branches (Yang et al. 2012: figs 1, 25, 30). All Jurassic to Cretaceous Osmylopsychopidae and Psychopsidae have the recurrent veinlet (e.g., Peng et al. 2010: fig. 3A; VM, pers. obs.). Some species of the Middle Jurassic to Early Cretaceous family Kalligrammatidae possess the well-developed recurred veinlet (e.g., Yang et al. 2009: fig. 3, Yang et al. 2011: figs 2, 3A, Ponomarenko 2002: fig. 254); however, this condition is obviously absent in other species, even from the Middle Jurassic (VM, QY, pers. obs.).

The costal space in all numerous undescribed Ithonidae from the Middle Jurassic locality of Daohugou is basally narrowed, and therefore, the recurrent veinlet (where well developed) has short simple branches. Sometimes, the humeral veinlet is not branched and crossvein-like (VM, pers. obs.). Younger Ithonidae (Early Cretaceous to Recent) have the costal space usually broader basally and have a well developed recurrent veinlet (e.g., Archibald and Makarkin 2006: figs 5B, 8D, 9A; Makarkin and Archibald 2009: fig. 3; Makarkin et al. 2012: figs 3D, E). All species of the Middle Jurassic to Early Cretaceous subfamily Mesomantispinae possess a rather well developed recurred veinlet (Makarkin 1996: fig. 1; Jepson et al. 2013: figs 1C, 2D, 4B). A similar recurred veinlet is present in the extant subfamily Symphrasinae, the single fossil species of which is known from the middle Eocene of Messel, Germany (Wedmann and Makarkin 2007). Other Mantispidae (both fossil and extant) do not possess the recurred veinlet. All fossil Hemerobiidae, including the oldest known specimens from the Late Jurassic and Early Cretaceous have well-developed recurred veinlet (e.g., Henriksen 1922: fig. 4; Panfilov 1980: fig. 91; Makarkin 1991: fig. 2a; Ponomarenko 1992b: fig. 4). Of the Berothidae, the well-developed recurrent veinlet is detected in the Late Jurassic to Early Cretaceous Mesithoninae (Panfilov 1980: figs 86, 87, 90; Ren and Guo 1996: fig. 6; Makarkin 1999: figs 2a, b, 4), and few other genera (e.g., *Plesiorobius* Klimaszewski & Kevan, 1986: fig. 2). In other berothids, the recurrent veinlet is poorly developed, only slightly recurrent and bearing few branches, or does not develop at all (e.g., Engel and Grimaldi 2008: figs 19, 21, 24, 25).

In the Mesozoic family Brongniartiellidae, the well-developed recurred veinlet with long branches is detected in two species from the Early Cretaceous of the Baissa locality, Transbaikalia (Makarkin 2010: fig. 3). The taxonomic affinities of several Mesozoic genera which bear a well developed recurrent veinlet are uncertain, e.g., *Sibithone* Ponomarenko, 1984: fig. 5a (Early / Middle Jurassic of Novospasskoe, Siberia); *Osmylogramma* Ponomarenko, 1992b: fig. 5 (Early Cretaceous of Tsagan-Tsab, Mongolia); *Meilingius* Ren et al., 2002: fig. 2 and *Jurapolystoechotes* Ren et al., 2002 (both from Daohugou; pers. obs.).

**Figure 7.**
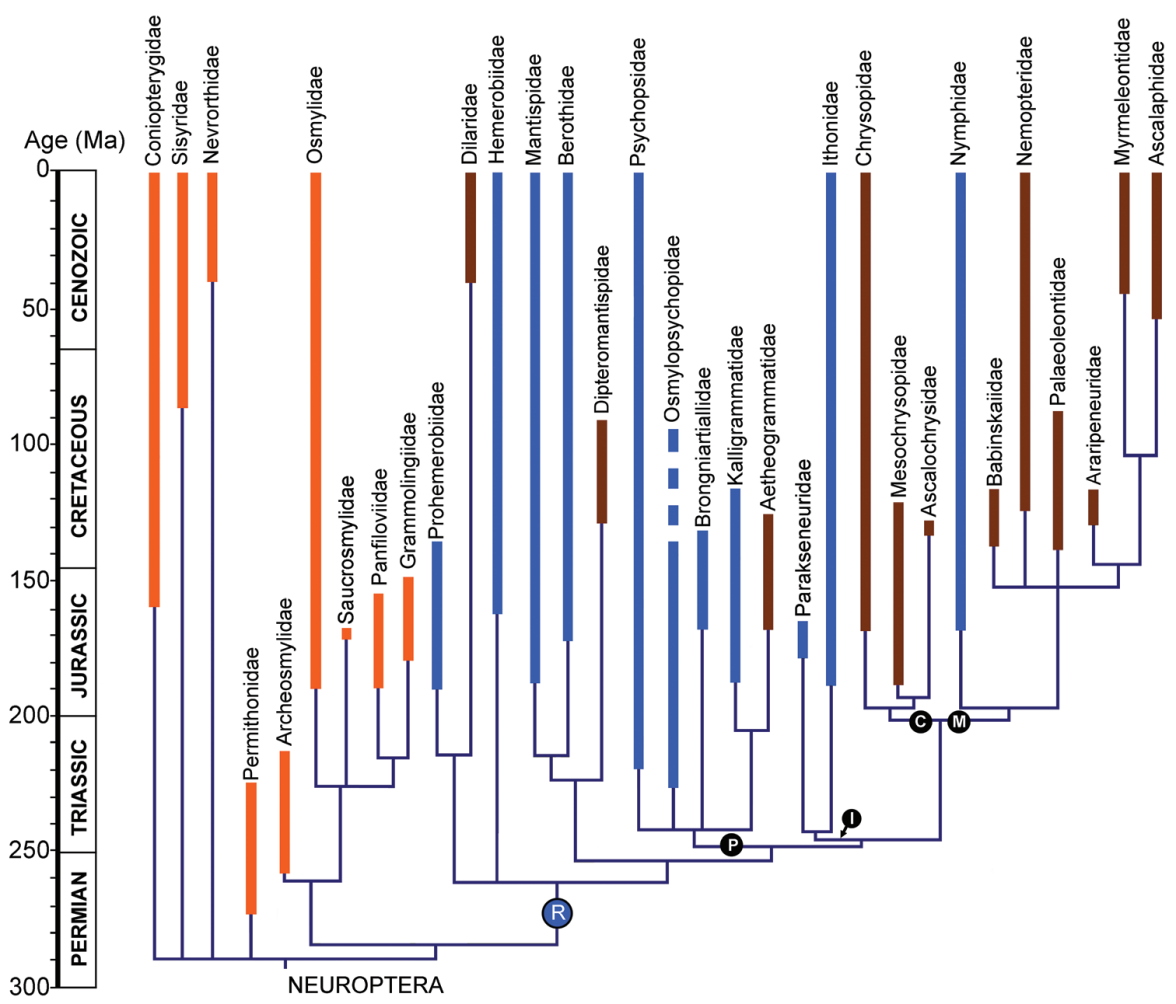
The occurrence of the recurrent veinlet through Neuroptera families. The phylogeny of the order is based on that of Yang et al. (2012), but modified according to the most probable relationships of families as discussed by Yang et al. (2012, 2013) and Makarkin et al. (2013).The families that we propose originally lacked the recurrent veinlet are represented in orange; R, families possessing the recurrent veinlet in blue, and those that we propose lost it secondarily in brown. **I** the ithonoid clade; **C** Chrysopoidea (the chrysopoid clade); **M** Myrmeleontoidea (the myrmeleontoid clade); **P** Psychopsoidea (the psychopsoid clade).

The presence of the recurrent veinlet was long considered a plesiomorphic condition in Neuroptera (e.g., MacLeod 1970; Oswald 1993; Aspöck and Nemeschkal 1998) because the most ancient, ‘primitive’ extant families possess this condition (e.g., Ithonidae, Psychopsidae), and but it is absent in the youngest, more derived families (e.g., Myrmeleontidae, Ascalaphidae). More recently published phylogenetic analyses of the group place these families in more derived positions and thus the presence of the recurrent veinlet is now considered an apomorphic state evolving independently at least three times (Winterton et al. 2010; Winterton and Makarkin 2010; Yang et al. 2012). The origin of the recurrent veinlet throughout Neuroptera most likely occurred during in the late Permian to earliest Triassic (Figure 7). This condition was secondarily lost at least five times in those families of the clade ‘R’ that lack this condition; and was never possessed in families basal to this clade.

The two species described above from the Middle Jurassic locality of Daohugou are the oldest known record of Nymphidae, indeed, of Myrmeleontoidea. Myrmeleontoidea are believed to belong to the suborder Myrmeleontiformia, which also includes Psychopsidae (Aspöck et al. 2001) or all families of Psychopsoidea (Engel and Grimaldi 2008). According to our phylogeny (Yang et al. 2012: fig. 32), however, the clade comprising Myrmeleontoidea and Chrysopoidea is sister to the ithonoid clade, and that combined clade is sister to the psychopsoid clade. Known psychopsoid fossils are much older than those of Myrmeleontoidea, and the recurrent veinlet first appears in their Triassic representatives (see above). Therefore, the presence of the recurrent veinlet in the Middle Jurassic Nymphidae may be interpreted as a remnant of ancient condition occurring in the psychopsoid + ithonoid + chrysopoid + myrmeleontoid clade before separation of the clade Myrmeleontoidea + Chrysopoidea. This character condition was lost in other families of Myrmeleontoidea and Chrysopoidea, and in the younger Nymphidae.

The recurrent veinlet is known mainly in species whose costal space is basally dilated. The strong dilation of the basal portion of the costal space is characteristic of Neuroptera. It never occurs in other Holometabola including other Neuropterida orders, although in some taxa, the costal space is markedly dilated at some distance from wing base (e.g., Raphidioptera: Aspöck 1986; the mecopteran family Dinopanorpidae: Archibald 2005: figs 3A–D). The costal space of earlier Neuroptera (basal to the clade ‘R’) is also narrowed basally. The appearance and development of the recurrent veinlets and the strong dilation of the basal costal space are probably functionally and structurally tied. However, in some Mesozoic Neuroptera which possess the recurrent veinlet the costal space is narrowed basally: the two species of the Middle Jurassic Nymphidae described here, and at least some Early Jurassic Prohemerobiidae and the Middle Jurassic Ithonidae. It seems reasonable that these taxa probably retained the recurrent veinlet after the costal space was secondarily narrowed in those lineages.

### Subcosta anterior (ScA)

The ScA in all Neuroptera (when present) has a similar configuration: a short vein structure terminating on ScP before the recurrent veinlet. The structure of ScA of *Daonymphes bisulca* gen. et sp. n. is very similar to that found in extant Nymphidae, especially *Nymphes* Leach, 1814 (VM, pers. obs.) and closely related genera (e.g., *Austronymphes* sp.: see Riek 1967: fig. 3D). A similar ScA occurs in most extant Hemerobiidae (e.g., Makarkin 1993: fig. 13). Other extant Neuroptera have no distinct ScA, but a convex sclerotized bulge presented in the humeral area of most other families is considered as the modified ScA (Kukalová-Peck and Lawrence 2004). Of the fossil taxa, the ScA is present in the Mesozoic Kalligrammatidae (e.g., Yang et al. 2011: fig. 2) and Parakseneuridae (Yang et al. 2012: figs 24, 25, 30; QY, VM, pers. obs.). According to the hypothesis of Kukalová-Peck (1983), the ScA of the pterygote wing venation ground plan is a relatively short vein running to the costal margin with a strong subcostal brace connecting ScA and ScP midway (Kukalová-Peck 1983, fig. 15), or by other interpretation the ScA is divided midway into the anterior branch (ScA1+2) running to the costal margin and the posterior branch (ScA3+4) terminating on ScP (Kukalová-Peck 1991: fig. 6.3C). Such a primitive ScA is believed to present in the gigantic Carboniferous Bojophlebiidae (Riek and Kukalová-Peck 1984: fig. 20; Kukalová-Peck 1991: fig. 6.14A), although it appears to be poorly preserved (Prokop et al. 2010). In general, the configuration of ScA in some Paleozoic Palaeoptera is similar to that of Neuroptera in that ScA is terminating on ScP, i.e., in Dictyoneuridae (Palaeodictyoptera; see Kukalová 1970: figs 55, 59, 60, 73) and some families of Ephemeroptera (e.g., Carpenter 1979: figs 1, 8, 11); however, the ScA is branched in the latter order.

The ground-plan Neoptera wing venation is hypothesized to have ScA consisting of two separate veins with no common stem, ScA1+2 and ScA3+4; the former runs to the costal margin, the latter is terminated on ScP (Haas and Kukalová-Peck 2001: fig. 1; Kukalová-Peck 2009: fig. 14). Therefore, the ScA of Neuroptera may be interpreted as the homologue of this hypothesized ScA3+4, i.e., as the ground-plan neopteran ScA lost its anterior branch ScA1+2. ScA in other Neuropterida orders (Megaloptera, Raphidioptera) is configured similarly to most other winged insects, i.e., a vein terminating on the costal margin (Kukalová-Peck 1983: fig. 17A; 1991: fig. 6.16; Liu et al. 2013). Such a configuration of ScA is most developed in Orthoptera (e.g., Béthoux and Nel 2001: fig. 1). This state of ScA may be interpreted as the homologue of the hypothesized ScA1+2, i.e., as the ground-plan Neoptera ScA lost its posterior branch ScA3+4.

The configuration of ScA characteristic of Neuroptera (i.e., short and terminating on ScP) does not occur in other orders of Neoptera, and therefore may be considered as an autapomorphy. On the other hand, the similarities of ScA in Neuroptera and some Paleozoic Palaeoptera (see above) may indicate their homology, and therefore a symplesiomorphy. The latter, however, appears to be less probable than the former.

## Supplementary Material

XML Treatment for Daonymphes

XML Treatment for Daonymphes bisulca

XML Treatment for Liminympha

XML Treatment for Liminympha makarkini
